# Sulfonated Polyether Ketone Membranes Embedded with Nalidixic Acid—An Emerging Controlled Drug Releaser

**DOI:** 10.3390/polym15173631

**Published:** 2023-09-01

**Authors:** Himabindu Padinjarathil, Vidya Vilasini, Rajalakshmi Balasubramanian, Carmelo Drago, Sandro Dattilo, Prasanna Ramani

**Affiliations:** 1 Dhanvanthri Laboratory, Department of Sciences, Amrita School of Physical Sciences, Amrita Vishwa Vidyapeetham, Coimbatore 641112, India; 2 Department of Chemical Engineering and Materials Science, Amrita School of Engineering, Amrita Vishwa Vidyapeetham, Coimbatore 641112, India; 3 Institute of Biomolecular Chemistry, CNR, via Paolo Gaifami 18, I-95126 Catania, Italy; carmelo.drago@cnr.it; 4 Institute for Polymer, Composite and Biomaterials, CNR, via Paolo Gaifami 18, I-95126 Catania, Italy; 5 Center of Excellence in Advanced Materials and Green Technologies (CoE–AMGT), Amrita School of Engineering, Amrita Vishwa Vidyapeetham, Coimbatore 641112, India

**Keywords:** polyether ketone, sulfonation, nalidixic acid sodium, physicochemical studies, drug release kinetics, mathematical model

## Abstract

The effective administration of medication has advanced over decades, but the medical community still faces significant demand. Burst release and inadequate assimilation are major drawbacks that affect wound healing efficiency, leading to therapy failure. The widespread application of polymers in biomedical research is significant. The polyether ether ketone (PEEK) family is known for its biocompatibility, inertness, and semi-crystalline thermoplastic properties. In our present studies, we have chosen a member of this family, polyether ketone (PEK), to explore its role as a drug carrier. The PEK backbone was subjected to sulfonation to increase its hydrophilicity. The response surface methodology (RSM) was used to optimize the sulfonation process based on the time, degree of sulfonation, and temperature. The PEK polymer was sulfonated using sulfuric acid at 150 °C for 6 h; back titration was performed to quantify the degree of sulfonation, with 69% representing the maximum sulfonation. SPEK and nalidixic sodium salt were dissolved in dichloroacetic acid to create a thin membrane. The physiological and morphological properties were assessed for the SPEK membrane. The studies on drug release in distilled water and a simulated body fluid over the course of 24 h revealed a controlled, gradual increase in the release rate, correlating with a mathematical model and demonstrating the zero-order nature of the drug release. Hemolysis on the SPEK membrane revealed lower toxicity. The SPEK membrane’s biocompatibility was established using in vitro cytotoxicity tests on the Vero (IC_50_: 137.85 g/mL) cell lines. These results confirm that the SPEK membranes are suitable for sustained drug release.

## 1. Introduction

Pharmaceutical research and development have taken giant steps leading to modifying drug delivery systems that are therapeutically more efficient and innocuous [1,2,3,4]. In a conventional drug delivery system, the intake of the medical formulation via any route (commonly oral) causes the leaching of the drug into the body fluid medium as per the burst mechanism [5]. Such a sudden drug release can result in the improper assimilation of the therapeutic agent into the biological system. Maintaining optimum concentrations attained by controlled drug release as the time increases to achieve the therapeutic outcome remains challenging [5]. Polymers were employed to create devices for regulating drug distribution to replace failing natural organs during the early 2000s [6,7]. In the pharmaceutical world, the polymer has been recognized to have a significant role as a carrier, releaser, and controller in wound management and oral administration as coatings, binders, flavor maskers, protective agents, medication carriers, and release regulators, which are a few methods polymers are used to have faster degradability [3]. In matrix or membrane materials for transdermal patches, polymers are used as backings, adhesives, or medication carriers using biodegradable polymers as possible alternatives to deliver proteins and peptides under controlled conditions [8,9]. 

**Figure 1:**
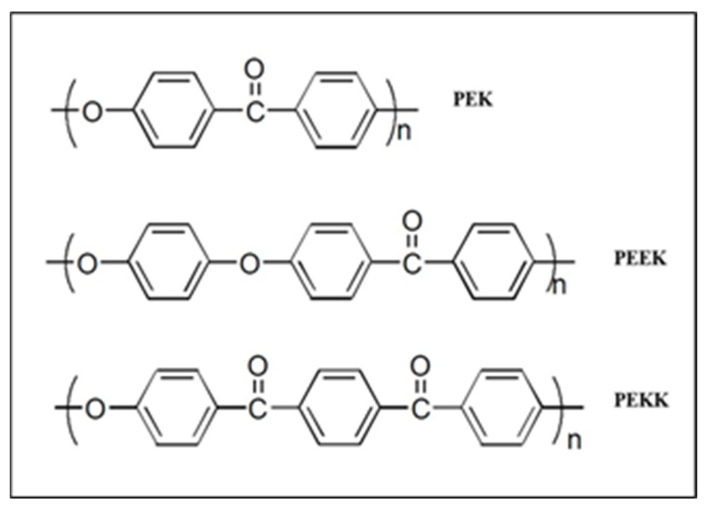
Structures of PEK, PEEK, and PEKK.

Polyaromatic semi-crystalline thermoplastics called PAEKs (polyaromatic ether ketones) are well-known for their usage in biomedicine, particularly as implants, surgical instruments, cardiovascular devices, and scaffolds for bone replacements; composites are used as microwave-absorbing materials [8,10,11,12]. Polyether ether ketone (PEEK), polyether ketone ketone (PEKK) (Figure 1) [13], and polyether sulfone (PES) [9] are examples of certain members showing features like superior biocompatibility and chemical inertness which demonstrated no cytotoxicity, mutagenicity, or carcinogenicity [14,15]. Polyether ketones are good candidates for medical components, subsea equipment, and valve components due to less degradability and inertness [15]. The sulfonation of PEK is used to improve the hydrophilicity of the hydrophobic polymer backbone. The process of sulfonation is chosen over nitration or hydroxylation because it is a simple, less time-consuming, and cost-effective procedure [15,16]. After a thorough literature review, it was determined that sulfuric acid would be the best sulfonating agent for this purpose. The response surface methodology is a powerful statistical model for optimizing the relationship between multiple variables and responses of interest. It is a valuable tool used to optimize the degree of sulfonation by considering various factors and their interactions to achieve polymer modification [17,18].

Nalidixic acid is an antibacterial and antimicrobial drug widely used to treat urinary tract infections caused by Gram-negative bacteria [16,17,18,19]. In our current research, for drug release kinetic studies, nalidixic acid sodium was chosen due to its stability at room temperature and relative ease of procurement. In this study, we report the usage of sulfonated polyether ketone membranes for the sustained release of nalidixic acid sodium and the employment of RSM for the sulfonation of PEK.

## 2. Materials and Methods

### 2.1. Materials

The PEK polymer was obtained as a powder from Gharda Chemicals Pvt. Ltd. (MW 95,000–105,000 Da, Mumbai, India). Nalidixic acid sodium salt (CAS No: 3374-05-8) and sulfuric acid (98%) were purchased from Sigma Aldrich (Mumbai, India). The solvents *N, N*-dimethylformamide (DMF), dimethylsulfoxide (DMSO), *N*-methyl pyrrolidone (NMP), and Dichloroacetic acid (DCA) were obtained from Avra Synthesis Pvt. Ltd. (Hyderabad, India), Nice Chemicals Pvt. Ltd. (Ernakulam, India), and SRL Chemicals Pvt. Ltd. (Mumbai, India), respectively. The following instruments were used to carry out the experiments: Bruker Quantax 200 X-ray spectrophotometer (Berlin, Germany), Bruker (VERTEX70 spectrometer, Mumbai, India), TA Instruments Q100 differential scanning calorimeter (SDT Q600/Q20, USA), Carl Zeiss RA-ZEI-001 (Gemini 300), and Shimadzu UV-1900 (Japan; 200–600 nm).

### 2.2. Statistical Experimental Design of Sulfonation

The central composite design (CCD) of the response surface methodology was used for the statistical modeling and optimization of the parameters affecting the sulfonation of the PEK (RSM). The components of this design are a fractional factorial or complete factorial design with a central point of 6 and the cubic one (8) is another design, often a star shape with experimental points set apart from the center [17]. Three crucial factors of the sulfonation of the PEK (concentration of sulfuric acid, temperature, and time) are distinct variables, each at five levels (-α, -1, 0, 1, and α, where α =1.68179). Referring to equation N = K^2^ + 2K + Cp, where K is the total factors and Cp is replicating multiple central point trials [18,20]. We could use quadratic models because we chose low and high range limits for each parameter [20]. Table 1 lists the types and levels of the independent control variables examined in this study of the concentration of sulfuric acid, temperature, and time.

The plan for a central composite design (CCD) to conduct the experiments is shown in Table 2. The CCD plan considers the response as the degree of sulfonation calibrated by the titration method. It should be emphasized that the amount of polymer (1 g) and the reaction performed in the nitrogen condition are kept constant during sulfonation. The RSM graphs were plotted to analyze the experimental data using regression and to find the best conditions for sulfonation of the PEK using the statistical software package Design Expert from Stat-Ease 360 version 22.0.5.0.

### 2.3. Synthesis of Sulfonation PEK (SPEK)

About 1.8 g of PEK and 36 mL of concentrated H_2_SO_4_ (98%) were taken in a 100 mL single-necked round bottom flask and agitated/mixed continuously for 24 h at room temperature. The brown-colored solution obtained was then transferred into ice-cold deionized water, and the solid substance was filtered and rinsed using deionized water to remove the excess sulfuric acid. The SPEK was then dried overnight at 50 °C in a hot air oven [21,22,23]. The sulfonation process was optimized by varying four parameters, such as the time, temperature, solvents, and concentration used. Of the different sulfonating solvents tested, sulfuric acid was deemed the most suitable for the reaction with respect to yield, cost, feasibility, and ease of reaction. To determine the optimum reaction temperature, the reaction was carried out at 10 °C, 25 °C, 80 °C, 100 °C, 120 °C, 150 °C, and 185 °C. The reaction time (1 h to 72 h) and reagent concentration and the polymer-to-acid ratios 1:10, 1:20, 1:25, 1:50, and 1:500 were tested. The reaction duration was another parameter that was tested at regular intervals, ranging from 15 min to 72 h. The quenching of the reaction was performed using 3–4 mL of reaction mixture over a wide range of time intervals. Each quenched solid sample was filtered, washed, dried, and used for further analysis, like a solubility test, DoS determination, and FTIR analysis.

### 2.4. Characterizations of SPEK

#### 2.4.1. Degree of Sulfonation by IEC

About 50 mg of the PEK was weighed and soaked in a predetermined (0.1 N) solution of NaCl for a minimum of 24 h. The polymer solution was titrated using phenolphthalein as an indicator against standard NaOH [24]. The result was triplicated using the same key. The titer value was inserted in a simple formula to determine the degree of sulfonation of the polymer. To ensure the completion of the ion exchange process, another titration was carried out after 48 h to verify that there is a degree of sulfonation despite the variation in the soaking period [25].

(1) $$DOS(\%)=\frac{Molecular\;weight\;of\;monomer\times IEC\times 100}{1000-\left(IEC\times 80\right)}$$

(2) $$IEC=\frac{V_{NaOH}\times N_{NaOH}}{W}$$

where IEC is the ion exchange capacity of the sulfonated polymer. DOS is the degree of sulfonation of the polymer. V_NaOH_ is the consumed volume of the NaOH in the experiment. N_NaOH_ is the NaOH normality, determined by the standardization titration against the standard oxalic acid solution. W is the weight of the polymer soaked in the standard NaCl solution. The molecular weight of the monomer is 196.21 g/mol.

#### 2.4.2. Chemical Interaction of SPEK

The FTIR and ^1^H-NMR analyses were performed to confirm the sulfonation reaction. The thermogravimetric analysis (TGA) was performed in a nitrogen gas atmosphere to determine the thermal stability, glass transition temperature (Tg), and changes in the onset of decomposition of the SPEK membranes. The SPEEK membranes’ morphology was investigated via a field-emission scanning electron microscope. Five different membrane samples were tested.

### 2.5. Membrane Casting

The SPEK (0.5 g) was dissolved in 4 mL dichloroacetic acid (DCA) and 2 mL dichloromethane (DCM) and heated at 150 °C for a period of 1 h in a round-bottomed flask fitted with a water condenser [26,27,28,29]. The resulting suspension was poured into two petri dishes in equal amounts and allowed to cool to RT. Subsequently, the colloid was washed with deionized water until the pH was neutral to remove the excess DCA used. Nalidixic acid sodium (7 mg) was dissolved in the DMF (0.5 mL) added to one of the petri dishes and mixed thoroughly to obtain a uniform suspension. The petri dishes without the drug and with the drug were kept in a vacuum oven at 50 °C for a period of 16 h, which yielded thin membranes.

### 2.6. Water Uptake Studies

The SPEK membrane was submerged in deionized water for performing the water up-take studies. The swollen membrane (after drying) was weighed in regular intervals of time [24,25,27]. The weight of the SPEK membrane before the study was noted. The percentage of water absorption was calculated by plotting a graph using the aforementioned data in the equation given below.

(3) $$Water\;absorption\;percentage=\frac{W_{s}-W_{i}}{W_{i}}\times 100$$

where W_s_ is the swollen weight and W_i_ is the initial dry weight.

### 2.7. Pharmaco-Physicochemical Studies on SPEK Membrane

The distribution coefficients of the drug molecules in the simulated body fluid (SBF)–polymer membrane system was considered by employing the amount of drug released after 24 h and the total amount of drug loaded into the membrane. The diffusion coefficients of the polymer-SBF system can be determined by the equation of Fick’s law of diffusion [29,30]. It can be determined using lag time, which is the shortest time the system takes to attain the sustained release of the active ingredient. The lag time and thickness of the membrane can be used to determine the diffusion coefficient by using the following equation:

(4) $$D=\frac{h^{2}}{6t_{L}}$$

where D indicates the diffusion coefficient of the polymer membrane, h indicates the thickness of the polymer membrane, and t_L_ is the lag time in the system before a sustained release is observed (one hour in our case).

In accordance with Overton’s rule, the distribution and diffusion coefficients equation was used to obtain the permeability coefficient:

(5) $$P=\frac{KD}{∆x}$$

P is the polymer membrane’s permeability coefficient, K is the drug’s distribution coefficient in the polymer water system, D is the polymer’s diffusion coefficient, and x is the thickness of the membrane when it is utilized with and without the drug.

### 2.8. In Vitro Drug Release Studies from SPEK Membrane

The drug release kinetics were examined by employing the UV-Vis absorption method [28,31,32]. The SPEK membrane loaded with nalidixic acid sodium salt was immersed in a predetermined amount of the SBF. The system was then subjected to periodic agitation, and the solution was withdrawn and subjected to a UV–Vis Absorption Spectroscopy measurement. Deionized water was used as a blank. All plots and other graphs were drawn by using UV absorbance at 331 nm (nalidixic acid sodium salt). By comparing the absorbance at various time intervals with that of reference drug solutions, the amount of drug released into the solvent system was estimated. The drug distribution coefficient in the polymer/SBF system was estimated from these valves.

### 2.9. Mathematical Models

Drug release is a significant aspect of pharmacokinetics and is a prerequisite for therapeutic agent absorption and a factor in the rate and extent of active availability in the body. Often, a mathematical equation can be found to express the dependence of release as a function of time [33,34]. We have selected the mathematical models which are applicable to membranes as our study was focused on drug-loaded membranes. The Hopfenberg model and Ritger–Peppas model were used to study the mechanism of release of the drug [34].

### 2.10. Hemolysis

A photometrical analysis was used to understand the hemolysis of the membranes. The blood samples collected were first processed by removing the plasma and leukocytes using the Ficoll-hypaque density gradient centrifugation method [35]. The pellet consisting of erythrocytes was washed thrice in phosphate buffer solution (PBS) and diluted (1:9). To investigate the impact of DCA, the polymer PEK, SPEK, along with the SPEK membrane (after water washing, A-SPEK, and without water washings, B-SPEK) were incubated in 0.8 mL of PBS at 37 °C for 30 min. About 0.2 mL of diluted erythrocytes was added and incubated for an additional two hours and centrifuged at 1600 rpm for 10 min. The absorbance of the supernatants was measured at 570 nm in the UV spectrophotometer to know about the free hemoglobin and lysed one (As) [36]. The erythrocytes with PBS were kept as the negative control (An) and water as the positive control (Ap).

(6) $$\%\;Hemolysis=\frac{As-An}{Ap-An}*100\;$$

### 2.11. In Vitro Biocompatibility Studies

The SPEK membrane was sterilized and subjected to the autoclave at 15 °C for 15 min, as the sterilization process. The stock solution was prepared in DMSO (32 mg/mL), and the working solution was prepared using DMEM plain media to dilute and attain the required concentration for the treatment process. Vero cell line cells were cultivated (5 x 10^4^ cells per well), seeded on 96-well plates in Dulbecco’s modified Eagle’s medium (DMEM, Sigma) with 10% FBS, and incubated at 37 °C. The epithelial cells were taken from an African green monkey. Confluent cells in the well were treated with a working solution at varied concentrations (25, 50, and 100 g/mL) after 24 h. Thereafter, the medium was replaced with fresh medium (1 mL), and MTT (6 mg/10 mL of MTT in PBS) was supplied to each well after the media had been incubated at 37 °C for 24 h. This was once more incubated for 4 h at 37 °C. After carefully draining the supernatant, 1 mL of DMSO was applied to each well. To determine the optical density (OD) value, the absorbance of the solution was calculated using a microplate reader (Bio-Rad 550) at 570 nm. The following expression was used in an experiment to determine the cell viability.

(7) $$\%\;Inhibition=\left[100-(\frac{OD\;of\;Sample}{OD\;of\;Control})\right]\times 100$$

### 2.12. Statistical Analysis

Three replicates’ means and standard deviations (SD) were used to express the results. One-way analysis of variance (ANOVA) was used to determine the statistical significance (5%) and was followed by a Student’s t-test with a *p* value of 0.05. The GraphPad Prism programme, version 5.0 (GraphPad programme, Inc., La Jolla, CA, USA), was used for all statistical analyses.

## 3. Results and Discussion

### 3.1. Statistical Optimization of Sulfonation of PEK

Response surface methodology (RSM) has been the most popular modeling and optimization approach in recent years. The sulfonation of polymers like PEEK has been optimized for nanofiber synthesis to enhance proton conductivity using the RSM [37]. This study examined the influence of processing factors on the degree of sulfonation of PEK determined by the back titration method with a constant amount of polymer and using continuous factors: the concentration of sulfuric acid, temperature, and time (Table 2).

The highest-order polynomial was selected which showed significance when compared to other models, like linear, quadratic, etc. (Table S1, Supporting information). The adjusted R^2^ and the predicted R^2^ were obtained for each response assessment using the analysis of variance (ANOVA) of the quadratic model. ANOVA was performed to calculate the REs and the coefficient of determination for the model results to examine the performance of the models (R^2^) (Table S2, Supporting informaiton). The *p*-value, which was smaller than 0.0001, determines each coefficient in the model’s significance. If the *p*-value is equal to 0.001, it signifies the coefficient’s importance; the model is suggested to be significant by the model F-value of 25.08. An F-value this big might happen owing to noise just 0.01% of the time. The model terms are considered significant if the *p*-values are less than 0.0500. The models A, B, C, AB, and BC are significant in our study; the predicted R² of 0.8838 differs by more than 0.2 from the adjusted R² of 0.2893, which might point to a significant block impact. Therefore, the significant model was considered in our current research. Poly(ether ether ketone) (PEEK) was analyzed by the parameter by using the design of experiments analysis [38]. The nozzle temperature was found to be the most influential parameter on the tensile properties for printing the PEEK polymer which was determined via RSM analyses [39,40].

We have used least squares regression with normally distributed errors as the foundation for our analysis. The analyzed responses, which are listed in Table 1, underwent an analysis of variance (ANOVA). The complete quadratic model for the degree of sulfonation and the entire interaction model for the degree of sulfonation was significant based on the *p*-value, with all the factors stated in Table 2. The Table S3 in supporting information shows the coefficient estimate of the expected changes in the response per unit change in the factor value in response, assuming that all other variables stay constant. In an orthogonal design, the intercept is the average response over all runs. Based on the selections for the various components, the coefficients are modifications made to that average. The VIFs are 1 when the factors are orthogonal; VIFs higher than 1 show multicollinearity; and the higher the VIF, the more severe the correlation of the components. VIFs under 10 are generally considered acceptable. The following is the regression equation:

Degree of Sulfonation = 41.43972 − 1.77195 Concentration − 0.471816 Temperature − 0.960542 Time + 0.019075 Concentration * Temperature + 0.038814 Concentration * Time + 0.026797 Temperature * Time (8)

**Figure 2:**
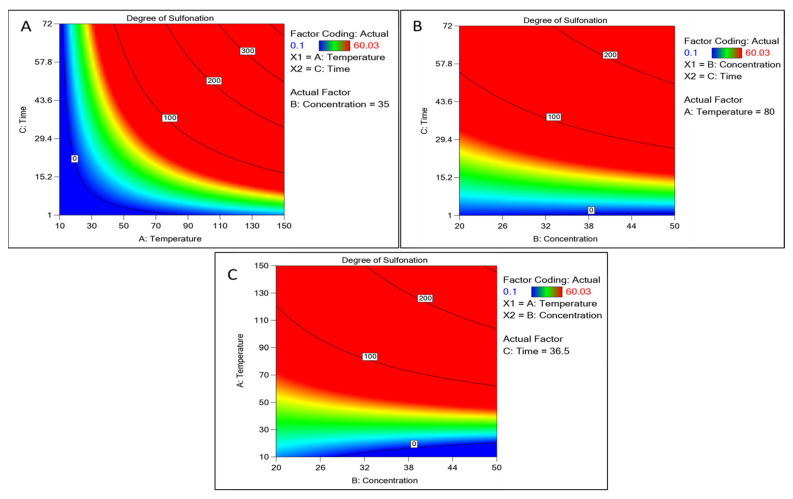
Contour plot of continuous factors like concentration of sulfuric acid, time, and temperature concerning the degree of sulfonation as a response. (**A**) Contour plot between time and temperature. (**B**) Time and concentration-based plots. (**C**) Temperature and concentration plot with the degree of sulfonation.

**Figure 3:**
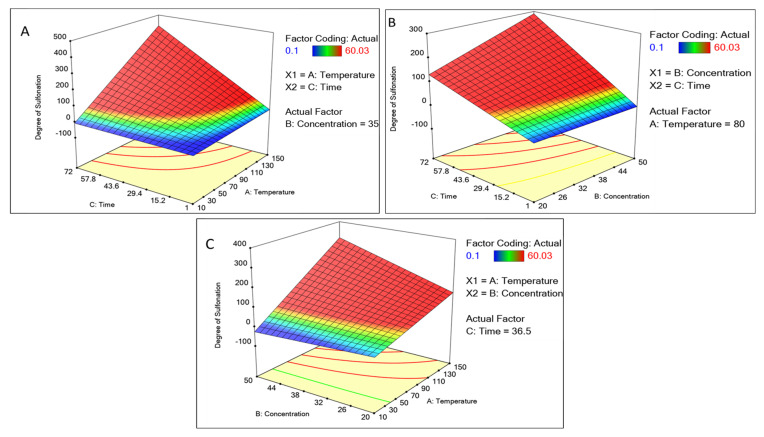
Surface 3D plot of continuous factors like concentration of sulfuric acid, time, and temperature concerning the degree of sulfonation as a response. For the degree of sulfonation. (**A**) Plotted with temperature and time. (**B**) Concentration and time, and (**C**) concentration and temperature.

The element may be predicted using the equation expressed in terms of the actual factors. Because the coefficients are scaled to account for the units of each element and the intercept is not at the center of the design space, this equation should not be used to estimate the relative importance of each factor. The concentration of sulfuric acid and time showed a positive correlation with an increase, whereas the temperature above 110 °C showed a decline (Figure 2). Other factors like the time interval showed increased sulfonation as the time increased. A graphical multi-response was used to analyze the factor’s effect on the degree of sulfonation. After superimposing the contour plots for all the responses, the regions that best satisfied all the requirements were chosen as the ideal circumstances. The maximum achievable sulfonation with the temperature, time, and concentration were the primary criteria for constraint optimization. Due to these restrictions, the best solutions fell into an “optimal region” (the shaded area in the overlay contour plots), Figure 2. The hold value of each continuous factor was obtained such as the temperature 80 °C, time 36 h, and concentration 35 mL which were considered the optimum and best. There is a common region for all the replies in the superimposed contour plots for the 3D production process. With the hold values, we plotted a 3D surface plot of the factors and responses, Figure 3. The surface plot also shows quadratic effects in two factors from the time, concentration, and temperature. The shape of such a surface is known as a “rising ridge” at the highest time and temperature, whereas the sulfuric concentration is less considered (Figure 3). Similar observations were reported for the optimization of the methyl ester sulfonation using the RSM [23].

Yuqing Zhang [39] worked on the sulfonation of PEEK using the quadratic models as a foundation, and a graphic of the multi-response optimization technique was used to identify the ideal parameters. In this report, the reaction concentration of 19 mL/g, reaction temperature of 60 °C, and reaction time of 3 h were observed to obtain the desired sulfonated polymer.

### 3.2. Sulfonation

Optimization of the sulfonation reaction was performed by varying the temperature, time, concentration, and solvent reaction environment. The presence of sulfonic acid groups was confirmed by FTIR and ^1^H-NMR (Figure S1). The degree of sulfonation ranged from 0.1% to ~69%. The maximum degree of sulfonation (69%) was obtained after 6 h of sulfonation time at 150 °C. A portion of the reaction mixture was quenched after 3 h to obtain SPEK with 20% of the degree of sulfonation. The SPEK displayed solubility in solvents, such as NMP, DMSO, and DMF.

By using a polymer-to-sulfuric acid ratio of 1:20, the resultant sulfonated PEK gave good yield; when the ratio was reduced to 1:10, it yielded low sulfonation on the polymer backbone. Poor reaction yield was obtained at high temperatures (above 150 °C), and the high ratio of PEK—H_2_SO_4_ (PEK: H_2_SO_4_ at 1:50 ratio) proved that these conditions were not suitable to meet our current objective. The SPEK was resistant to dissolving in most of the common organic solvents and was soluble in H_2_SO_4_ and DCA. The SPEK obtained at reaction conditions such as 150 °C and by using the 1:50 ratio (PEK: sulfuric acid) yield was very low; however, it demonstrated solubility in solvents, such as DMF, DMSO, and NMP. A higher yield of SPEK was obtained when the reaction was performed under a nitrogen gas atmosphere and a slightly higher degree of sulfonation when compared to the same reaction performed at the atmospheric condition in the absence of nitrogen. Lakshmana et al. worked on the syntheses of polyether ketone by the nucleophilic and electrophilic routes. They concluded that the high molecular weight of the polymer process and high melting point result in efficient thermal stability, solvent resistance, and a crystalline nature [40]. Sulfonation of the polymer was carried out with chlorosulfonic acid as the sulfonating agent, and it was observed that the good membrane of this polymer exhibited good permeability and hydrophilicity [29,41]. The sulfonated polymers, with a high degree of sulfonation, can swell or form emulsion and were found to be difficult to isolate [42]. In the reported literature, the SPEEK polymer showed degradation and elevated hydrophilicity as the sulfonation increased which is similar to the current investigation [43,44]. The polymer PEK’s insolubility and high melting temperatures impose processing and polymerization constraints [40]; due to this behavior, the PEK has a lower rate of sulfonation than the PEEK.

### 3.3. Solubility and Membrane Casting

The sulfonated PEK (SPEK) remained insoluble in *N*,*N*-dimethylformamide (DMF), *N*-methyl pyrrolidone (NMP), and dimethyl sulfoxide (DMSO); however, excellent solubility was observed for the PEK treated with H_2_SO_4_ (1:50 concentration ratio) at 150 °C for 3–6 h. The colloidal product was soluble in DMSO, DMF, and NMP, but the yield was significantly low, discouraging us from carrying out any further studies in this line. The research conducted by D. Liu et al. explains the insolubility of PEK in conventional polar reagents (like DMAc, CHCl_3_, and DMF) and the solubility of the polymer in dichloroacetic acid (DCA) [45]. Malik et al. used dimethyl acetamide (DMAc) as a solvent to prepare a polymer membrane by dissolving the SPEK in a solvent at 60 °C with constant stirring [42]. Our observations are similar to those of the reported ones. SPEK (48 h of sulfonation time at RT, 1:20 PEK:H_2_SO_4_) with an ~40% degree of sulfonation was used to cast a membrane with a combination of DCA and DCM as suitable solvents as per the reported procedure by Liu [36,44]. The amount of drug integrated into the drug delivery device system varies based on the substance, the intended therapeutic impact, and the release duration [34]. The drug-loaded membrane was prepared, taking 7 mg of nalidixic acid sodium as the initial drug concentration and mixing it with the dissolved SPEK in a petri dish.

### 3.4. Degree of Sulfonation

The time and ratio of reactants (PEK: H_2_SO_4_) are the other two variables that were observed to have a direct influence on the extent of the sulfonation, but after a specific juncture, a reversal in this relationship was observed in the reaction and led to the disintegration of the polymer. The degree of sulfonation was determined by the back titration method as per the reported procedure [24] and demonstrated a linear relationship with the temperature of the reaction which is in accordance with the reported literature [27,30]. With an increase in temperature (up to 150 °C), the degree of sulfonation also reached 70% (Table 2). Akhtar et al. reported that the degree of sulfonation of the SPEEK polymer was found to increase (from 3% to 46%) with an increase in the reaction time which was performed at room temperature [44]; a similar pattern of an increase in sulfonation was noted in our case for the SPEK (Table 3). A reaction with the polymer-to-sulfonating agent ratio set as 1:20 with a reaction time of 48 h (at 25 °C) gave a degree of sulfonation of ~40% which was chosen for our current study.

### 3.5. Characterization of SPEK

**Figure 4:**
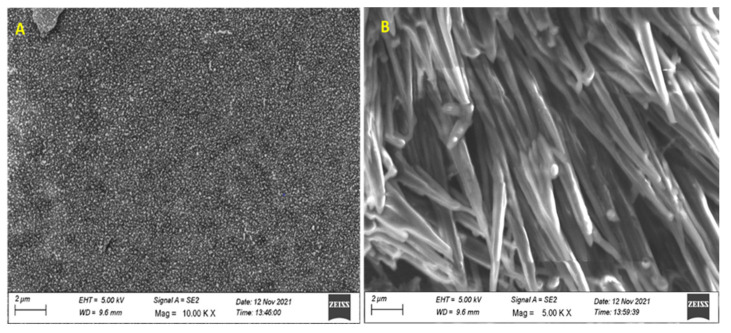
SEM images of SPEK membranes (**A**) without the drug and (**B**) with drug. Nalidixic acid sodium salt incorporated at 2 µm.

The membrane thickness was measured using a screw gauge and it was observed to have a homogeneous thickness (113 ± 0.3 μm). The surface morphology of the SPEK membranes was studied using SEM (Figure 4). Magnified images of the SPEK film showed prominent features of the homogenous distribution of the circular domain structure.

However, the drug-loaded film shows a phase-segregated morphology of coat spike rod-like structures. The non-homogeneity could be due to the solvent evaporation mode of membrane formation.

#### 3.5.1. FTIR Spectrum Studies

**Figure 5:**
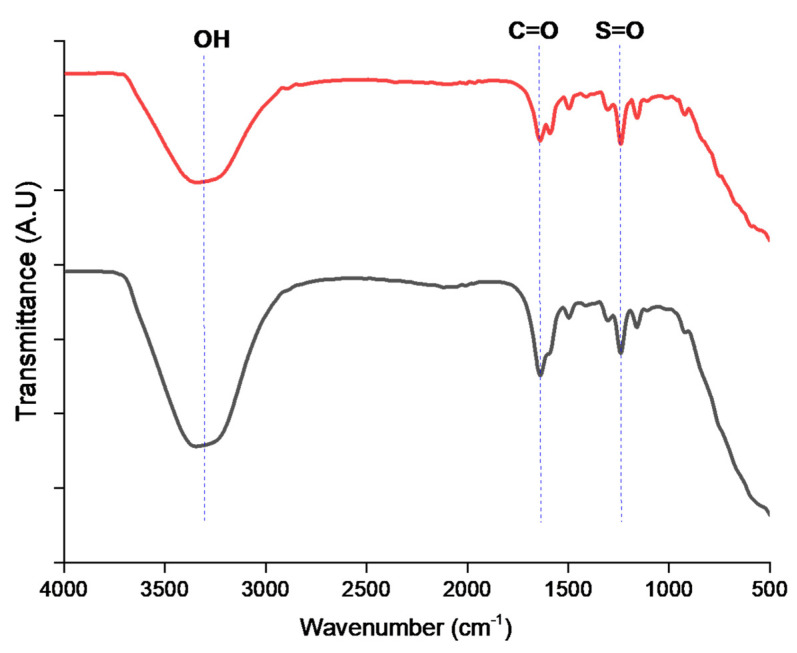
FTIR of low (red line) and high (black line) sulfonated PEK.

In the FTIR spectra obtained, a peak corresponding to carbonyl stretching was observed around 2000–1500 cm^−1^ (Figure 5). 

The peak corresponding to O=S=O vibration was observed around 1000–1250 cm^−1^, as reported by Sinan Feng et al. [30], thus confirming the presence of the sulfonic group. According to Feng, the aromatic ring between the ether groups has split broad peaks between 1471 and 1478 cm^−1^. The presence of sulfonic acid groups was observed in both low and high SPEK at 1250 cm^−1^ which corresponds to the S=O frequency. A broad band at 3250 cm^−1^ was observed which corresponds to –OH stretch. Our results are in line with the reported literature on a sulfonated brominated-PEEK polymer by Linder J Anderson et al. [26,27,31,44].

#### 3.5.2. TGA Analysis

**Figure 6:**
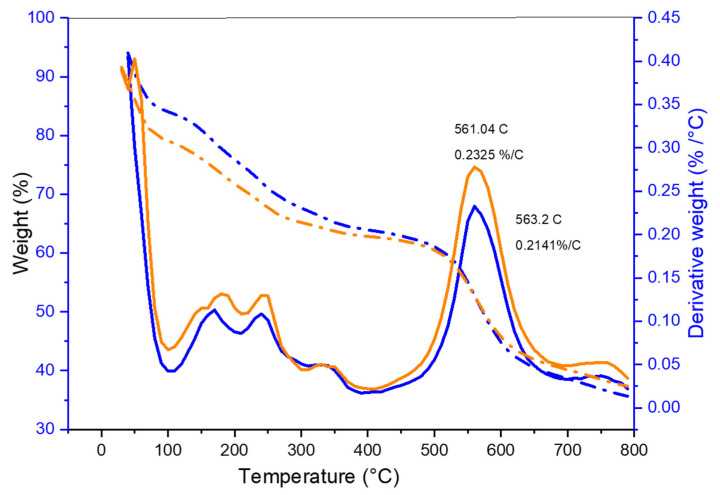
TGA and DTGA graph of SPEK membrane (orange color) and SPEK-NA membrane (blue color) loaded with the drug. The dashed lines indicate the derivative weight loss analysis TGA, and the straight line indicates the result of DTGA analysis in weight percentage loss.

A thermogravimetric analysis was carried out under a nitrogen atmosphere using a shallow platinum crucible as a sample holder. The initial weight loss was observed at 100 °C which was related to water loss, during the film processing, which may have been trapped in their matrix, as seen from both thermograms. This observation correlates with the FTIR of the –OH stretch which corresponds to the hydrated membranes. The more considerable weight loss that followed was caused by the ejection of sulfur trioxide from the polymer matrix as a result of the breakdown of the sulfonate groups that were introduced into the polymer matrix during the sulfonation process (Figure 6). As a result, polymeric amine counter ions stopped the sulfonic acid’s negative charge, which is extremely susceptible to hydrolytic heat degradation. The breakdown of the polymer chain can be linked to progressive weight loss [44,46]. Because the large loss in weight of the sample is more frequently detected when the temperature breaches the barrier of 650 °C, the temperature at which the analysis was capped in this case, although some loss in the weight of the sample was recorded, was less significant. Although in very small amounts, the decomposition product at 450 °C can be attributed to the polymer chain breaking down into 4-phenoxy phenol, and the steady reduction up to 650 °C led to the formation of carbon monoxide and carbon dioxide, which in our case was then arrested using the stoppage of the analysis [25,47]. The Tg values for the PEK are almost between l00 and over 200 °C, while the Tm value is 341 °C.

### 3.6. Water Uptake Studies

**Figure 7:**
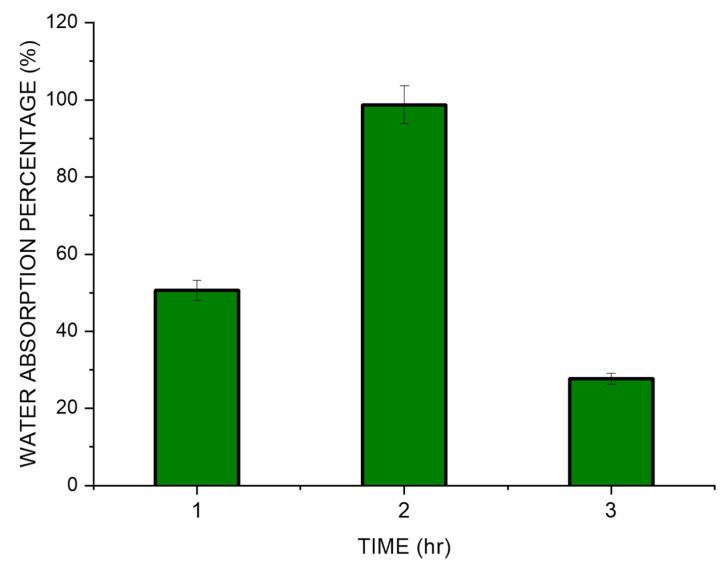
Water uptake studies in SPEK membrane.

The SPEK membranes were soaked in deionized water to understand the update of the water by the polymer film and was observed to have a maximum of 98.7% water by weight after the initial 2 h (Figure 7). After a period of two hours, slow disintegration of the membrane was seen. We have followed the research conducted by Sinan Feng et al. which showed the evidence of a water uptake study being conducted with PEK [30]. A study by Chen et al. showed that sulfonated Cardo PEK (S C PEK) displayed an increase in the uptake of water as there was an increase in the degree of sulfonation [24]. According to them, sulfonic acid groups help the membrane to swell and assist in the penetration of water. However, excess water absorption may lead to excessive swelling, mechanical fragility, and morphological instability of the membrane. The sulfonation of thermoplastic polymers, notably ion exchange membranes, can experience a period of initial water uptake followed by a negative water uptake value. This is due to the high hygroscopicity of the sulfonic groups that in the intact membrane retain a small percentage of water (due to humidity, for example) which cannot be easily eliminated by heating. During the experiment with the deterioration of the membrane, it becomes easier to completely dry the material [48]. Sulfonated polyether ether ketone (SPEEK) membranes, which are widely utilized in fuel cells and other electrochemical applications, frequently exhibit this behavior. The release of water that the membrane had previously absorbed is what causes the negative water absorption that is seen after the initial phase. A sulfonated thermoplastic polymer membrane’s unique behavior can change based on elements, including the level of sulfonation, polymer structure, membrane thickness, and environmental circumstances [16,28,49].

### 3.7. Drug Release Kinetics

**Figure 8:**
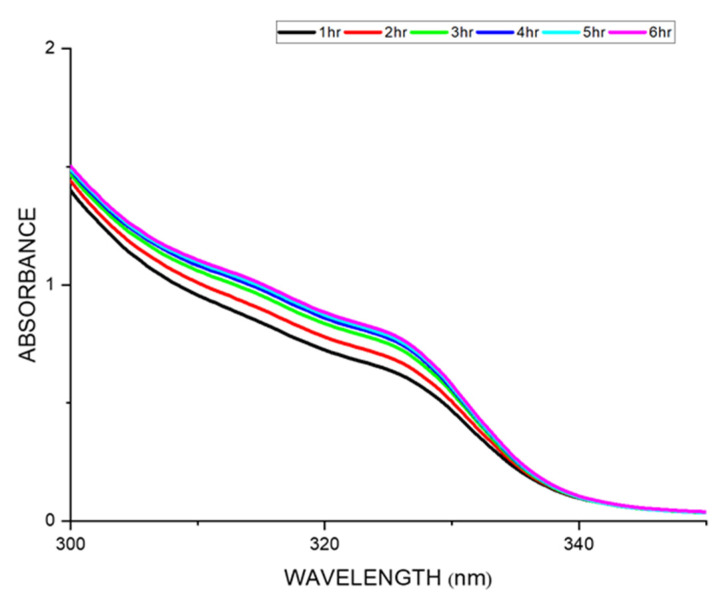
Drug release studies of SPEK membrane loaded with nalidixic acid sodium for 6 h.

The drug release kinetics of a drug-loaded membrane were studied by UV absorbance analysis by submerging the drug-loaded membrane in deionized water for a period of 24 h [34]. The plot between the absorbance vs. wavelength showed the drug release when the wavelength was swept across various time intervals for the nalidixic acid sodium salt-loaded sulfonated membrane (Figure 8). The absorption maximum for the nalidixic acid sodium salt is 331 nm, and there was a gradual increase in the absorption value during the initial six hours, after which it remained constant for a successive 24 h. In general, membranes formed through the solvent evaporation technique show the diffusion mechanism of drug release. The erosion or swelling of polymers could be due to osmotic processes, ion exchange, or other diverse phenomena, if the polymer is hydrophilic in nature [6]. The incorporation of the drug in a hydrophobic or hydrophilic polymer matrix is a common design to achieve a controlled drug release mechanism. In our study, we have observed that the release of the drug happens via the diffusion control mechanism. The erosion mechanism is ruled out in our case as the values are less significant (Table S4, Supporting information) which is in accordance with the literature report [47,48].

### 3.8. Distribution Coefficient, Diffusion Coefficient, and Permeability Coefficient

The amount of drug released at the end of 24 h and the total amount of drug put into the membrane were used to calculate the distribution coefficient of the nalidixic acid sodium salt in the SBF–polymer membrane system (with a 40-percent degree of sulfonation). It was observed that 4.185 was the distribution coefficient. The diffusion coefficients of the SBF–polymer system were calculated using the equation of Fick’s law of diffusion and were determined to be 5.911 × 10^−7^ cm^2^/s. The permeability coefficient of the nalidixic acid sodium salt-loaded SPEK membrane was calculated to be 2.19 × 10^−4^ cm/s.

### 3.9. Mathematical Models of Drug Release

The drug release kinetics were studied by applying zero-order kinetics and the Hopfenberg and Ritger–Peppas models [31,33]. As time progressed, the drug release rate of the system was seen to be independent of the concentration of active ingredients in the matrix. When the concentration of the drug-loaded one was lowered, there was no variation or decrease in the drug release rate which indicates the zero-order nature of the drug release. The results from the Hopfenberg model reveal that the drug release occurred primarily due to diffusion and not the erosion mechanism (10^−6^ cm/s) which correlates with the experimental drug release studies. The Ritger–Peppas drug release model demonstrates the drug release fits to the Fickian diffusion model (Table S4, Supporting information).

### 3.10. Hemolysis Studies of SPEK

**Figure 9:**
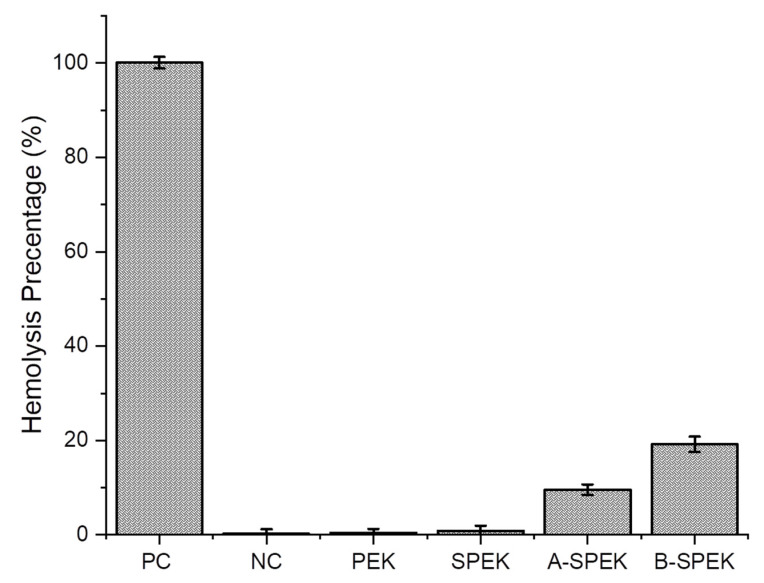
Hemolysis studies. A-SPEK: SPEK after water washing; B-SPEK: SPEK without water washing.

It is essential to understand the level of toxicity on the sulfonated PEK membranes, as they encounter blood directly when applied as a bandage or indirectly when injected into the body [35]. Hemolysis studies were performed in human erythrocytes treated with PEK, SPEK, and water-washed SPEK as well as SPEK without water washing (after DCA treatment). The lysis due to the membrane was analyzed using spectrophotometry. Low hemolytic activity (<5%) was observed by PEK and SPEK, but a slightly higher hemolysis (7%) was seen on the SPEK washed with water (Figure 9). We have used deionized water and PBS as the positive (PC) and negative controls (NC). In the case of B-SPEK, a high level of hemolysis (54%) was observed due to the highly acidic conditions in the membrane consisting of the trapped residues of DCA, while in the case of A-SPEK (9.5%). From this observation, it is evident that the water washings reduce the concentration of DCA present in the membrane which in turn makes less hemoglobin spillage and erythrocyte lysis leading to it being less hemolytic and having high compatibility. In addition, this analysis proved that the sulfonated PEK has lower toxicity which correlates with the literature [49,50].

### 3.11. In Vitro Cytotoxicity Studies of SPEK

**Figure 10:**
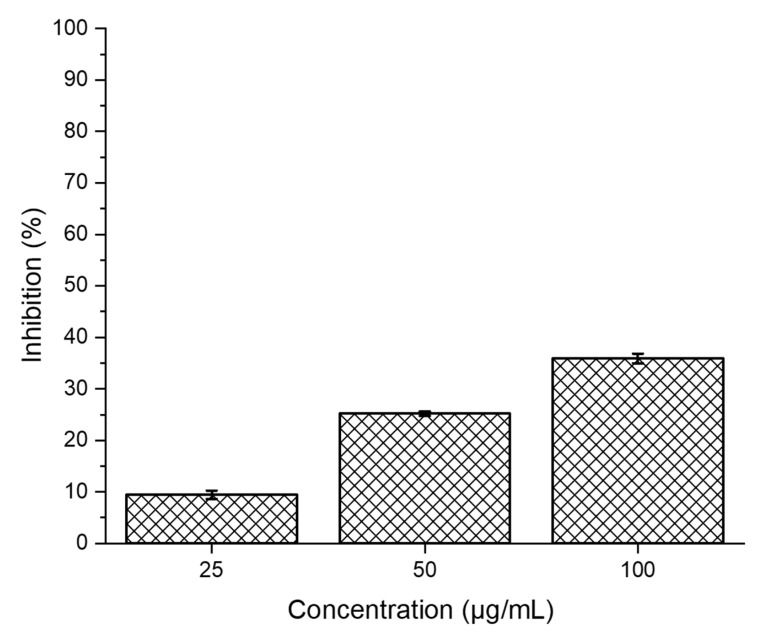
The MTT assay VERO cell lines at three different concentrations after 24 h.

The cytocompatibility of SPEK was studied against a Vero cell line for a period of 24 h (mammalian continuous cell line) to establish its biocompatibility nature. From Figure 10, as the concentration increases, we have noted a slight increase in cytotoxicity. The IC_50_ of the SPEK membrane was found to be 137.85 µg/mL which is less significant, proving that the membrane is biocompatible in nature. Similar reports are available in the reported literature [51].

## 4. Conclusions

The PEK was subjected to sulfonation to increase its hydrophilicity. The response surface methodology was used to optimize the sulfonation process based on the level of sulfonation and other parameters; a maximum degree of sulfonation (69%) was achieved. The membranes of the SPEK and SPEK with nalidixic sodium salt were obtained with uniform thickness and characterized for their physical, physiological, and morphological properties. The studies on drug release in deionized water and simulated body fluid over the course of 24 h revealed a regulated, gradual increase in the release rate, linking it with a mathematical model and demonstrating the zero-order nature of the drug release. The hemolysis studies on the SPEK membrane showed low toxicity which is in line with the in vitro cytotoxicity of the SPEK membrane on the Vero cell lines (IC_50_ of 137.85 g/mL), indicating that the membranes are not affecting the normal cells to a greater extent. These results show that the SPEK membranes could be used as a medium for sustained drug release.

## Figures and Tables

**Table 1:** Experimental independent factors and levels of variables in RSM.

Code	Factors	Low	High
A	Concentration of sulfuric acid (mL/g)	20	50
B	Temperature (°C)	10	150
C	Time (h)	1	72

**Table 2:** RSM design by CCD and observed responses.

Entry	Temperature (°C)	Concentration (mL/g)	Time (h)	Degree of Sulfonation (%)
1	10	20	1	0.10
2	10	20	2	0.28
3	150	50	6	60.03
4	10	20	6	0.25
5	10	20	24	5.50
6	10	20	48	10.62
7	10	20	72	12.00
8	30	50	24	8.62
9	30	20	1	5.43
10	30	20	2	6.77
11	100	50	6	27.79
12	30	20	6	16.9
13	30	20	24	24
14	30	20	48	37
15	30	20	72	42
16	150	50	3	33.14
17	100	20	1	1.18
18	100	20	3	2.5
19	100	20	6	8.00
20	100	50	1	3.50

**Table 3:** Optimization of SPEK reaction.

S.no	Polymer: H_2_SO_4_	Temperature (°C)	Time (h)	Degree of Sulfonation (%)
1	PEK (1:20)	10	1–72	0.10–10.95
		RT	1–72	3.43–40.02
		100	1–72	4.75–57.00
		150	1–6	4.63–15.89
2	PEK (1:50)	RT	1–24	3.45–8.00
		100	1–6	7.00–37.95
		150	3–6	20.14–69.13
3	PEK (1:500)	RT	1	Failed

## Data Availability

The data presented in this study are available on request from the corresponding author.

## Supplementary Materials

The following supporting information can be downloaded at: https://www.mdpi.com/article/10.3390/polym15173631/s1. Table S1: Data showing the selection of model in RSM; Table S2: ANOVA for the 2FI model for response Degree of sulphonation in RSM; Table S3: Data showing the coefficients in terms of coded factors in RSM; Table S4: Data showing the First order, Zero order kinetic, Hopfenberg Model (Erosion Coefficient (m/h) and Ritger -Peppas model of SPEK membrane with Nalidixic acid sodium 1 to 6 hr period; Figure S1: 1H-NMR magnification of sample 2 obtained with the reaction at 100°C for 6 hr.
