# Supplementary material for: Sulfonated Polyether Ketone Membranes Embedded with Nalidixic Acid—An Emerging Controlled Drug Releaser
Source: Polymers (Basel). 2023 Sep 1;15(17):3631. doi: 10.3390/polym15173631 (PMC10490094; doi:10.3390/polym15173631)
Supplement: Supplementary file 1 [file polymers-15-03631-s001.zip › polymers-2498212-supplementary.pdf]

SUPPORTING INFORMATION

Sulfonated Polyether Ketone Membranes Embedded with Nalidixic Acid—An Emerging Controlled Drug Releaser.

Himabindu Padinjarathil [1,2], Vidya Vilasini [1], Rajalakshmi Balasubramanian [1], Carmelo Drago[3], Sandro Dattilo [4,*] and Prasanna Ramani [1,5,*]

[1] Dhanvanthri Laboratory, Department of Sciences, Amrita School of Physical Sciences, Coimbatore 641112, Amrita Vishwa Vidyapeetham, India
[2] Department of Chemical Engineering and Materials Science, Amrita School of Engineering, Coimbatore 641112, Amrita Vishwa Vidyapeetham, India
[3] Institute of Biomolecular Chemistry, CNR, via Paolo Gaifami 18, I-95126 Catania, Italy; carmelo.drago@cnr.it
[4] Institute for Polymer, Composite and Biomaterials, CNR, via Paolo Gaifami 18, I-95126 Catania, Italy
[5] Center of Excellence in Advanced Materials and Green Technologies (CoE–AMGT), Amrita School of Engineering, Coimbatore 641112, Amrita Vishwa Vidyapeetham, India
* Correspondence: sandro.dattilo@cnr.it (S.D.); r_prasanna1@cb.amrita.edu (P.R.)

Table S1: Data showing the selection of model in RSM

Response 1: Degree of sulphonation

Source	Sequential p-value	Lack of Fit p-value	Adjusted R^2	Predicted R^2	
**Linear**	0.0097		0.4066	0.1199	
**2FI**	**< 0.0001**		**0.8838**	**0.2893**	**Suggested**
**Quadratic**	**0.0004**		**0.9666**	**0.9079**	

Sequential Model Sum of Squares

Source	Sum of Squares	df	Mean Square	F-value	p-value	
**Mean vs Total**	4709.07	1	4709.07			
**Linear vs Mean**	2664.30	3	888.10	5.34	0.0097	
**2FI vs Linear**	**2237.44**	**3**	**745.81**	**22.89**	**< 0.0001**	**Suggested**
**Quadratic vs 2FI**	320.58	2	160.29	17.12	0.0004	
**Residual**	102.97	11	9.36			
**Total**	10034.36	20	501.72			

Table S2: ANOVA for the 2FI model for response Degree of sulphonation in RSM.

Source	Sum of Squares	df	Mean Square	F-value	p-value	
**Model**	4901.74	6	816.96	25.08	< 0.0001	**significant**
**A-Concentration**	202.45	1	202.45	6.21	0.0270	
**B-Temperature**	2027.73	1	2027.73	62.24	< 0.0001	
**C-Time**	1843.89	1	1843.89	56.60	< 0.0001	
**AB**	880.34	1	880.34	27.02	0.0002	
**AC**	146.40	1	146.40	4.49	0.0538	
**BC**	1114.78	1	1114.78	34.22	< 0.0001	
**Residual**	423.55	13	32.58			
**Cor Total**	5325.28	19				

Factor coding is **Coded**.
Sum of squares is **Type III – Partial**

Table S3: Data showing the coefficients in terms of coded factors in RSM

Factor	Coefficient Estimate	df	Standard Error	95% CI Low	95% CI High	VIF
**Intercept**	87.86	1	9.80	66.68	109.04	
**A-Concentration**	17.56	1	7.05	2.34	32.78	22.85
**B-Temperature**	82.17	1	10.42	59.67	104.68	30.35
**C-Time**	90.23	1	11.99	64.32	116.14	37.06
**AB**	20.03	1	3.85	11.70	28.35	2.56
**AC**	20.67	1	9.75	-0.3955	41.73	39.77
**BC**	66.59	1	11.38	42.00	91.18	31.63

Table S4: Data showing the First order, Zero order kinetic, Hopfenberg Model (Erosion Coefficient (m/h) and Ritger -Peppas model of SPEK membrane with Nalidixic acid sodium 1 to 6 hr period.

			**Zero order kinetic/ First order**	**Ritger -Peppas**	**Hopfenberg Model**
**Time (mins)**	Amount of drug released (mg)	Amount of drug left in t*60e membrane (mg)	fi=1-(Wi/Wo)	n=mi/mo/ko/t	mt/mo=1-[1-k0t/CoAo)n
**60**	0.95	6.05	$3.76*10^{-5}$	1	$5.96*10^{-6}$
**120**	1	6	$1.98*10^{-5}$	1	$3.14*10^{-6}$
**180**	1.1	5.9	$1.45*10^{-5}$	1	$2.3*10^{-6}$
**240**	1.15	5.85	$1.14*10^{-5}$	1	$1.8*10^{-6}$
**300**	1.15	5.85	$9.12*10^{-6}$	1	$1.44*10^{-6}$
**360**	1.15	5.85	$7.6*10^{-6}$	1	$1.2*10^{-6}$
**1260**	1.35	5.65	$2.556*10^{-6}$	1	$4*10^{-7}$
**1320**	1.35	5.65	$2.43*10^{-6}$	1	$3.9*10^{-7}$
**1380**	1.35	5.65	$2.32*10^{-6}$	1	$3.7*10^{-7}$
**1440**	1.35	5.65	$2.223*10^{-6}$	1	$3.5*10^{-7}$

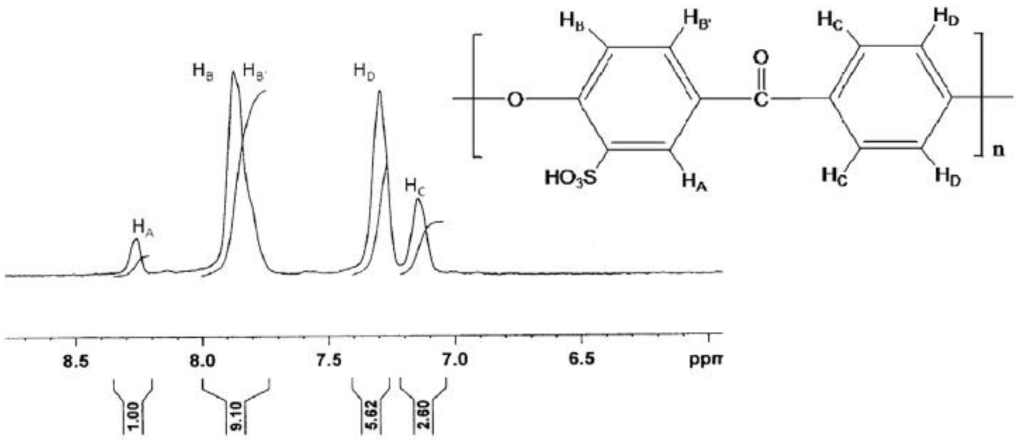

Figure S1: ^{1}H-NMR magnification of sample 2 obtained with the reaction at 100°C for 6 hr.
